# Elimination of *Legionella* colonization in a hospital water system: evidence from 23 years of chlorine dioxide use

**DOI:** 10.1017/ice.2025.25

**Published:** 2025-04

**Authors:** Natalie G. Exum, Lindsay N. Avolio, Gregory Bova, Clare Rock, Melanie S. Curless, Lisa L. Maragakis, Kellogg J. Schwab

**Affiliations:** 1 Department of Environmental Health and Engineering, Johns Hopkins Bloomberg School of Public Health, Baltimore, MD, USA; 2 Facilities Engineering, Johns Hopkins Medicine, Johns Hopkins University School of Medicine, Baltimore, MD, USA; 3 Department of Medicine, Division of Infectious Diseases, Johns Hopkins University School of Medicine, Baltimore, MD, USA; 4 Armstrong Institute for Patient Safety and Quality, Johns Hopkins University School of Medicine, Baltimore, MD, USA; 5 Hospital Epidemiology and Infection Control, The Johns Hopkins Hospital, Baltimore, MD, USA; 6 AMRIC (AntiMicrobial Resistance and Infection Control), Health Service Executive, Dublin, Ireland; 7 Department of Medicine, Trinity College Dublin, Dublin, Ireland

## Abstract

A hospital water system colonized with *Legionella* bacteria (three of four buildings, with > 50% of positive samples) was able to reduce detections to <1% positivity in the long term only after ClO_2_ was iteratively added first to the cold-water and then hot-water systems followed by pipe replacements (n = 6835 total samples).

*Legionella* bacteria are the leading cause of drinking water disease outbreaks in the United States and are considered an emerging respiratory pathogen of concern.^1^ High-risk individuals^2^ are vulnerable to Legionnaires’ Disease (LD), a severe pneumonia infection, in inpatient hospital settings where hospital-acquired infections account for approximately 20% of all legionellosis cases.^3^

Hospital buildings have complex water systems and favorable conditions for the proliferation of *Legionella*, including intermittent occupancy and recirculating hot-water systems that result in reduced temperatures at distal sites. To reduce the risk of *Legionella*, the U.S. Centers for Medicare & Medicaid Services has required healthcare facilities to develop and implement water management programs (WMPs) since 2018.^4^ WMPs establish engineering controls and processes to minimize the colonization, growth, and transmission of biofilm-based bacteria which includes *Legionella*. Secondary disinfection is one aspect of a WMP that is often implemented when a healthcare facility needs to reduce *Legionella* colonization. Chlorine dioxide (ClO_2_) has been shown to significantly reduce *Legionella* colonization over the short term in hospital water systems.^5,6^ The long-term effectiveness of ClO_2_ to control for *Legionella* is less well understood and would inform development of WMPs in healthcare and long-term care settings.

Here we describe the long-term effectiveness of ClO_2_ as a secondary disinfectant used in the WMPs of multiple buildings on a large hospital campus. This study includes four inpatient buildings that conducted rigorous, routine environmental surveillance of *Legionella*. Trends of percent positivity were evaluated using routine water samples collected quarterly over a 23-year period.

## Methods

The secondary disinfection of a hospital water supply system was initiated in 2001 within a large, urban teaching hospital with 1050 inpatient beds. The campus has four inpatient buildings that have populations with increased risk factors for LD, including surgical, oncology, bone marrow transplant, and hemodialysis patients, as well as intensive care units and operating rooms. Construction of Buildings A, B, and C all pre-dated the installation of secondary disinfection while Building D was new construction designed to include continuous ClO_2_ treatment in both the cold- and hot-water systems. For Buildings A-C, a ClO_2_ system (Halox Inc., Bridgeport, CT and Pureline Treatment Systems, Bensenville, IL) was retrofitted to the piping systems on the cold-water intake from the public water supply. For the hot-water systems, that recirculate through continuous loops, ClO_2_ was injected after the hot-water converters. In 2019, a replacement of the main potable water loop of the hospital system, serving buildings A, B and C, began due to the age and condition of the piping. In Building B, the hot- and cold-risers to the patient care floors were replaced in 2012 as part of a larger building renovation. For Building A only, the water main source from the public supply was switched from a low-pressure to a high-pressure source that also supplied the other inpatient Buildings B-D.

Sink faucets were selected for routine, quarterly water sampling and collected as first-draw water samples directly from either the hot- or cold-faucets. Approximately 100 mL of water was collected into sterilized high-density polyethylene bottles with enough sodium thiosulfate to neutralize 20 ppm of chlorine. Samples were shipped at ambient temperature to a Centers for Disease Control and Prevention Environmental *Legionella* Isolation Techniques Evaluation certified laboratory within 24-hours. *Legionella* culture was conducted using a modified ISO method with both nonselective buffered charcoal yeast extract agar plates and selective plates. All culture plates were incubated for seven days in a humidified incubator at 36 +\−2 °C. Latex agglutination (Oxoid Limited, Basingstoke, UK) and direct fluorescent antibody staining were used to identify representative isolates of recovered *Legionella*. Water samples were analyzed using percent positivity using routine water samples only.

## Results

**Figure 1.**
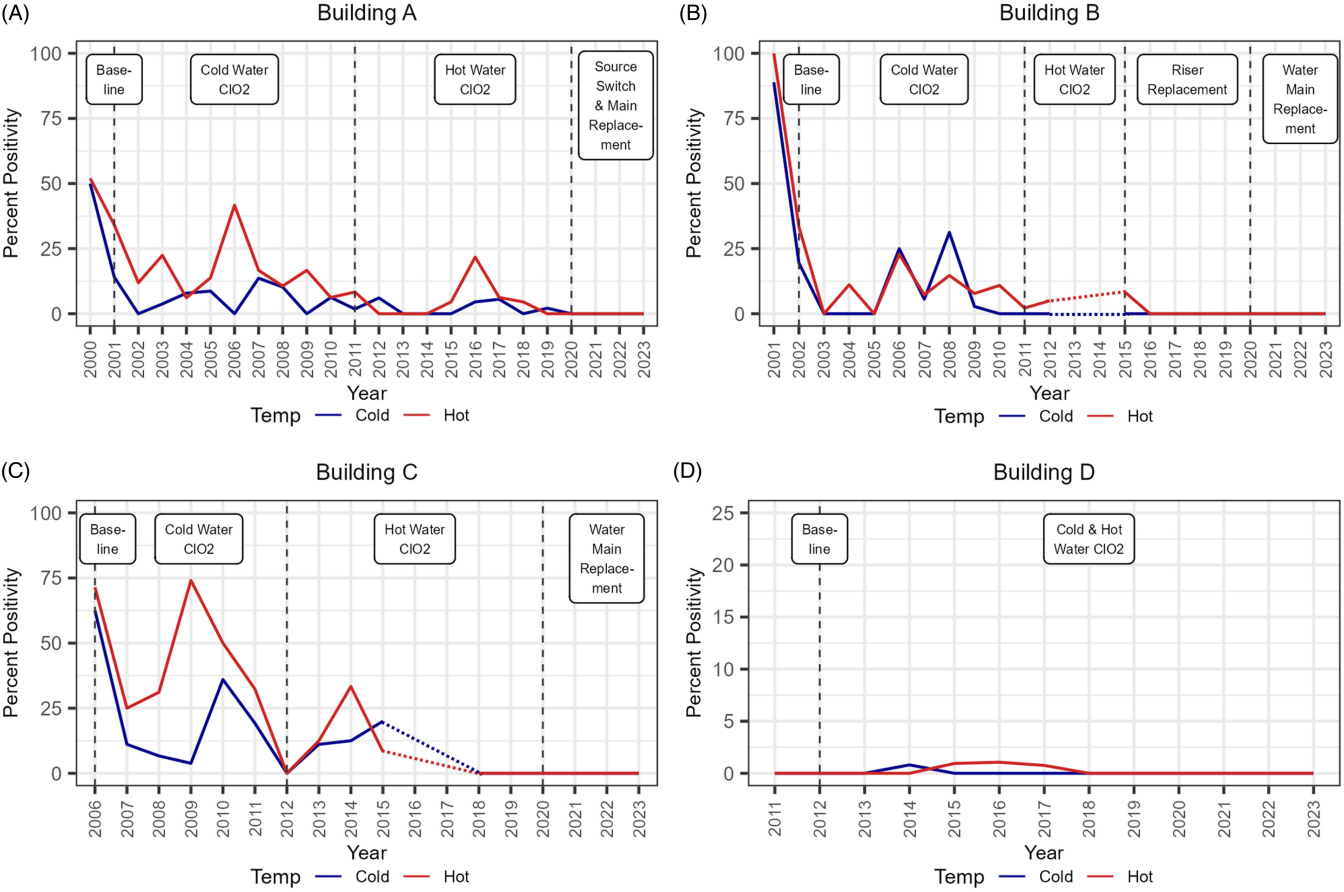
Annual percent *Legionella* positivity from water samples collected from hot- and cold-water sites. Interventions are represented by vertical dashed lines at the time of implementation within each building. Dashed temperature lines in (B) and (C) are inferred as no data was collected for these time periods due to building closure.

There were 6835 water samples included in this analysis of routine (quarterly) water sampling. The analysis of *Legionella* positivity from both hot- and cold-water sites shows a decrease over time within each Building A-C (Fig. 1). At baseline, at least 50% of both hot- and cold-water samples were positive for *Legionella* in Buildings A, B, and C, with no substantial difference in positivity between hot- and cold-water samples. After introduction of ClO_2_ treatment into the cold- and then hot-water systems consecutively, there were declines in percent positivity over time in both systems. Intermittent spikes in positivity of ClO_2_-treated water were found in Building A hot-water in 2016 (21.7%, 5 positive/23 total samples), Building B cold-water in 2008 (31.3%, 5 positive/16 total samples), and Building C cold-water in 2010 (36%, 9 positive/25 total samples) and hot-water in 2014 (33.3%, 3 positive/9 total samples). Riser and water main replacements in 2015 and late 2019 reduced the *Legionella* positivity down to consistently low levels (<1%) in Buildings A, B, and C. Building D was new construction with ClO_2_ treatment incorporated as part of the WMP from its inception, and it maintained low positivity from the beginning of monitoring in 2011 (Fig. 1).

## Discussion

This study showed the use of ClO_2_ as a secondary disinfectant in a hospital water supply can eliminate *Legionella* colonization over the long term. Colonization was continually reduced with each consecutive intervention of ClO_2_ treatment of the cold- and then hot-water systems, followed by pipe replacements. The WMP prioritized patient safety by using first-draw samples collected from inpatient rooms to assess sites with the greatest exposure risk.

Prior studies have shown the effectiveness of ClO_2_ to control *Legionella* colonization over shorter periods. Vicenti et al. 2019 used a four-year follow-up period to show colonization could be reduced to 18% positivity in hot water.^6^ Zhang et al. 2009 found that *Legionella* could be controlled (<10% positivity) within 18 months of ClO_2_ treatment in two different hospitals.^7^ Casini et al. 2008 found that samples exceeding a *Legionella* colonization threshold of 10^3^ colony-forming unit (CFU)/mL over a 5-year timeframe were reduced by 83.8% after ClO_2_ treatment.^8^

Pipe replacements are important components of a WMP in addition to secondary disinfection. Deficiencies in ageing pipes can promote an environment that is favorable for biofilm growth and *Legionella* proliferation along with reduced hot-water temperatures, and water stagnation. For prevention of healthcare-associated legionellosis a well-designed and frequently updated WMP is necessary that includes system maintenance, staff training, and routine water surveillance.^9^

Consistent with prior studies, our study found that the introduction of ClO_2_ treatment into the cold-water system did not adequately reduce *Legionella* positivity in the hot-water system.^10^ The increased age of recirculated hot water may have decreased the ClO_2_ residual to levels that were no longer effective at controlling *Legionella* growth.

Study limitations include the retrospective study design resulting in more inconsistencies of sampling events, locations, and collections methods than would be expected in a prospective design with more consistent data management practices. The culture-based method used for detection varied over the 23-years with three life-cycles of the International Organization for Standardization (ISO) standards published (ISO 11731:1998, ISO 11731-2:2004, and the current version, ISO 11731:2017). The current method includes both direct culturing methods and membrane filtration allowing for more flexibility depending on the nature of the water sample and expected bacterial load.

This study highlights that once *Legionella* bacteria are established within a hospital water system it requires iterative approaches, alongside secondary disinfection with ClO_2_, to be eliminated. It is critical that these approaches are developed by a multidisciplinary team of hospital infection preventionists and facilities water managers to achieve elimination.
